# Editorial Note: Tick-borne relapsing fever Borreliosis, a major public health problem overlooked in Senegal

**DOI:** 10.1371/journal.pntd.0014465

**Published:** 2026-06-29

**Authors:** The PLOS Neglected Tropical Diseases Editors

The *PLOS Neglected Tropical Diseases* Editors issue this notice to update the previously published Expression of Concern on this article [1,2].

Following the publication of the article and Expression of Concern [1,2], PLOS investigated concerns pertaining to the reported ethical approval and the article’s adherence to *PLOS Neglected Tropical Diseases*’ research ethics policies.

Specifically, the Materials and methods section of this article [1] states that between January and December 2016 blood samples were taken from febrile patients in Niakhar, Ngayokheme, Toucar, and Diohine, Senegal, that each patient who came for consultation with a fever was systematically eligible for inclusion in this study regardless of whether the patient’s temperature was taken at the clinic, and that a point-of-care clinical database containing the results of the molecular analyses made it possible to identify all causes of fever for each patient who visited these three health posts and/or the health center. The article does not report whether patients gave consent for their medical data to be used in the study.

The ethics statement reports that the Senegal National Ethics Committee for Health Research (CNERS) approved this study in the context of epidemiological surveillance of fever cases (statement number #0087).

The ethics approval number #0087 was also reported in other publications [3], [4, 5, retracted in 6], and document #00.87/MSP/DS/CNERS has been provided in response to ethics approval concerns raised with another article [7–9]. The seventh author provided documents #001380/MSP/DS/DER and #00081/MSAS/DGS/DS/CNERS for editorial review.

- Document #00.87 MSP/DS/CNERS is an ethics approval document issued by the Ministère de la Santé et de la Prévention of the République de Sénégal on June 02, 2010, for protocol SEN37/09 involving a study titled *“Identification des agents pathogènes responsables de fièvre au Sénégal (Projet IDEPATH) dans les sites suivants: Niakhar (Fatick), Mlomp (Ziguinchor), Banafassi (Kédougou) et Keur Momar Sarr (Louga).”* It grants a one year approval for a study aiming to identify pathogens responsible for fever at different sites in Senegal.
- Document #001380/MSP/DS/DER is an administrative authorization issued by the Ministère de la Santé et de la Prévention of the République de Sénégal on May 31, 2011, and provides a one-year extension to the ethics approvals for protocols SEN21/09 and SEN37/09: “*Identification des agents pathogènes responsables de fièvre au Sénégal.* *Réalisation de tests diagnostiques chez les maladies consultant dans les dispensaires de Dielmo, Ndiop, Niakhar, Mlomp, Bandafassi et Keur Momar Sarr.”*
- Document #00081/MSAS/DGS/DS/CNERS is an ethics approval document issued by the Ministère de la Santé et de l’Action Sociale of the République de Sénégal on June 04, 2012, and provides continued approval for the protocols SEN21/09 and SEN37/09 listed above. The document does not clarify the validation period and does not state an expiry date.

PLOS reviewed the explanation and documentation provided by the institution and concluded that the documents did not fully resolve the concerns. Specifically, documents #00.87 MSP/DS/CNERS and #001380/MSP/DS/DER expired in 2011 and 2012 respectively, and PLOS remains concerned that the ethics approval document #00081/MSAS/DGS/DS/CNERS issued in 2012 was no longer valid during the sample collection in 2016. In addition, the ethics approval documentation provided for editorial review does not appear to cover all collection sites reported in the article, and PLOS remains concerned about the lack of (reporting of) informed consent for participation in this study.

In light of the unresolved issues, the Expression of Concern stands.
